# Trends in Benzodiazepine and Z-Drug Prescriptions in Eastern China (2015–2021)

**DOI:** 10.31083/AP38792

**Published:** 2025-02-28

**Authors:** Qian Deng, Hualiang Chen

**Affiliations:** ^1^Department of Pharmacy, The Seventh People’s Hospital of Shaoxing, 312000 Shaoxing, Zhejiang, China

**Keywords:** benzodiazepines, Z-drugs, outpatient and emergency prescriptions, trend analysis

## Abstract

**Objective:**

This study analyses trends in the prescription and usage of benzodiazepines (BZDs) and Z-drugs within specialised medical institutions and emergency outpatient services in China from 2015 to 2021, focusing on demographics and prescribing patterns to promote better management practices.

**Methods:**

A retrospective study was conducted from 2015 to 2021, reviewing prescription information and population characteristics from 10 hospitals, including specialised psychiatric institutions and general hospitals in Zhejiang, Jiangsu, and Shanghai. The study analysed a total of 33,569 valid prescriptions.

**Results:**

There was a noticeable increase in the total defined daily doses of both benzodiazepines and Z-drugs, with significant variations among different drugs. Lorazepam and zopiclone showed the most substantial increases in usage. Drugs like clonazepam and lorazepam were predominantly prescribed, indicating specific patterns in disease management, particularly for insomnia and anxiety.

**Conclusions:**

This study reveals a significant increase in benzodiazepine and Z-drug prescriptions, particularly among elderly and female patients. The findings highlight the need for targeted interventions and policy reforms to ensure safe prescribing practices and mitigate the risks associated with long-term use in these vulnerable populations.

## Main Points

1. Increasing Trend in Drug Usage: Our analysis indicates a consistent year-over-year increase in the prescription and use of benzodiazepines (BZDs) and Z-drugs in specialised medical institutions and outpatient settings, underscoring a growing reliance on these medications.

2. Demographic Insights: The study reveals significant demographic disparities in drug usage, with higher prescription rates among elderly female patients. This suggests a targeted need for monitoring and potentially adjusting prescription practices for this demographic.

3. Drug Safety Concerns: Our findings highlight safety concerns related to the increased use of potentially addictive medications among older populations. This calls for enhanced oversight and the implementation of stricter prescribing guidelines to prevent misuse and dependency.

4. Regional Variations in Prescriptions: The study documents notable differences in prescription trends across different regions, reflecting varying medical practices and accessibility to healthcare services. This emphasises the need for region-specific strategies to optimise drug use.

5. Implications for Policy and Practice: The results advocate for policy reforms and educational initiatives aimed at promoting the rational use of BZDs and Z-drugs to mitigate risks and improve patient outcomes.

## 1. Introduction

Benzodiazepine receptor agonists (BZRAs) are currently the most commonly used sedative-hypnotic and anti-anxiety drugs. This class includes traditional benzodiazepines (BZDs) and non-BZD Z-drugs. Benzodiazepines primarily produce their effects by binding to benzodiazepine receptors on the gamma-aminobutyric acid (GABA) receptor complex, enhancing the effect of GABA and leading to sedation and hypnotic, anticonvulsant, and muscle relaxation effects [1]. Z-drugs, although similar in function to BZDs, are primarily used for their sedative–hypnotic properties and are not typically considered anxiolytics. Most BZDs are internationally classified as Class IV controlled drugs and can cause serious social harm if acquired via illicit channels [2]. Benzodiazepines bind to specific sites on the GABA-A receptors in the brain and enhance the effects of GABA. This leads to increased chloride ion influx into the neurons, which hyperpolarises the cell membrane and reduces neuronal excitability [3]. Z-drugs are structurally different from BZDs but act on the same GABA-A receptor complex as BZDs [4]. They bind to a specific site on the receptor complex that is distinct from the benzodiazepine site. Benzodiazepines are the most commonly abused type of prescription drug, according to the World Health Organization (WHO) Global Drug Report 2018 [5]. A previous study examined the prevalence and prescribing patterns of BZDs and Z-drugs in older nursing home residents across six European countries [6]. Another study analysed trends in the consumption rates of BZDs and Z-drugs in the health region of Lleida, Spain, from 2002 to 2015 [7]. The current article focuses on the drug use trends of BZDs and Z-drugs in regional specialised medical institutions in China, which is a developing country. The study aims to address an existing knowledge gap by providing more specific and detailed information on the drug-use trends of BZDs and Z-drugs in rapidly developing areas, such as China’s East Coast, which differ from general hospitals or community settings in developed countries.

In recent years, the use of BZDs has been on the rise, and their excessive and irregular use has become a new topic [8]. The excessive and irregular use of BZDs has been investigated in countries around the world, revealing significant dependence and adverse effects risks. Previous study reveals that while benzodiazepine use is common among US adults, misuse and use disorders are relatively rare, though certain demographics are at higher risk for misuse and associated health issues [9]. Corresponding studies in China have shown similar patterns, highlighting the need for stricter regulation and better management practices to mitigate these risks [10]. This study aims to understand Zhejiang Provincial Hospital’s guidelines for the prescription of BZDs, as well as the selective BZD drugs that are used in this clinical setting. It also aims to analyse the prescription drug types, dosage, and quantity, and to understand the population characteristics of those who use prescription drugs. This will provide a scientific basis for the rational use of these drugs. The significance of this study is to present a better understanding of the actual situation behind the increase of prescriptions; the variety, dosage, and quantity of prescription drugs; and the structural factors, such as the gender, age, and disease of patients who use prescription drugs. This study aims to guide the rational use of drugs in the corresponding population and reduce drug abuse. By identifying trends in prescription practices and highlighting demographic groups at higher risk, our findings provide a basis for targeted interventions, such as educational programmes for healthcare providers and the implementation of stricter prescribing guidelines. The purpose of this study is to better understand the usage patterns of BZDs, which have a high potential for addiction, and to promote their rational use. By analysing prescription data based on gender, age, and disease factors, we aim to identify trends and improve management practices. Our findings are based on a sample from China’s eastern coastal regions, which are economically developed and have a higher usage rate of BZDs due to an ageing population [11].

## 2. Purpose

This study aims to understand Zhejiang Provincial Hospital’s guidelines for the prescription of BZDs, as well as the selective BZD drugs that are being used. This includes examining factors such as dosage, frequency, and patient demographics to ensure that prescriptions are made according to the best practices outlined in the hospital’s guidelines. It also aims to analyse the prescription drug types, dosage, and quantity, and to gain a better understanding of the population characteristics of those who use prescription drugs. The outcomes aim to provide a scientific basis for the rational use of these drugs.

## 3. Method

### 3.1 Research Design

This pharmacoepidemiology retrospective study reviewed prescribing information and population characteristics from 2015 to 2021. The study utilised a stratified random sampling approach, selecting data from 10 major hospitals in Zhejiang, Jiangsu, and Shanghai. This method ensured a representative sample across different types of hospitals, including specialised psychiatric institutions and general hospitals, thereby providing a comprehensive view of benzodiazepine and Z-drug prescription trends.

### 3.2 Institutions

This study focused on the use of BZDs and non-BZDs in major psychiatric hospitals in economically developed areas of eastern China from 2015 to 2021, as well as prescriptions for BZDs and non-BZDs in outpatient and emergency departments from 2017 to 2021.

### 3.3 Participants

The data of BZDs and non-BZDs in the hospital information system (HIS) refer to actual clinical usage and do not include data that are not actually used. Prescriptions for BZDs and non-BZDs used in outpatient and emergency prescriptions, with complete information and excluding unqualified returned prescriptions, were eligible for use in this study.

### 3.4 Variables

Defined daily dose (DDD) value: This refers to the average daily dose of medication. The DDD value is the total mean daily dose of the drug. The DDD values of each drug were queried and registered according to the DDD values published on the WHO’s website.

Prescription: This refers to an outpatient or emergency prescription issued by a registered physician.

Second-class mental prescriptions: In China, psychotropic drugs are divided into Class I and Class II psychotropic drugs. Class I psychotropic drugs are prescribed as special prescriptions for narcotic drugs, whereas Class II psychotropic drugs are prescribed with special prescriptions for Class II psychotropic drugs.

Drugs: These refer to BZDs and non-BZDs (class Z). The drugs include primarily BZDs, such as lorazepam tablets, oxazepam, estazolam tablets, alprazolam, clonazepam, diazepam needles, diazepam and nitrazepam tablets, while non-BZDs (class Z) include zopiclone tablets.

Diagnosis (name of disease): The names of mental diseases are classified according to the broad categories of related mental diseases denoted in the Treatment Guide: Psychosis Volume [7th edition] compiled by Australian Treatment Guidelines Limited, 2015. They include insomnia, sleep and anxiety disorders, and depression. Non-mental illness names are classified as ‘other diseases’.

### 3.5 Quantitative Variables

The DDD values of each drug were queried and registered according to the DDD values published on the WHO’s website.

Annual data were calculated and tabulated according to the drug name, specification, quantity, total measurement, and DDD number. Drug names were classified individually according to the prevailing chemical name of the drug.

Prescriptions: These were categorised by year, gender, age group, and drug name.

### 3.6 Data Sources and Measurements

Drug quantity: The quantity data of BZDs and Z-drugs that were used in relevant hospitals from 2015 to 2021 were collected, including the year, drug name, specification, unit price, and quantity. The data for this study were collected as part of a comprehensive research project on the prescription patterns of BZDs and Z-drugs in China. The study involved a total of 10 hospitals located in three provinces: Zhejiang, Jiangsu, and Shanghai. This included specialised psychiatric institutions and general hospitals with significant outpatient and emergency services.

Prescribed: The information system was based on the individual HIS of each hospital for drugs. Data were collected from 2017 to 2021 about the prescription and use of BZD drugs. The prescriptions contained no privacy information, as the names were displayed as ‘surname * *’. Patient data included their gender, age, diagnosis of diseases (name), drug name, quantity, usage, dosage, and prescription date. The collected data were checked for privacy and compliance.

### 3.7 Bias

The quantity of drugs refers to the actual consumption of said specific drug throughout the hospital as a whole.

Number of prescriptions: This was 8%–10% of the total sample collected for all 10 hospitals.

### 3.8 Statistical Methods

To ensure rigorous analysis and interpretation of the data collected, we employed a comprehensive statistical approach using the SPSS Statistics 21 software (IBM, Armonk, NY, USA). This section outlines the detailed statistical methods used in our study.

Data Preparation and Cleaning: Initially, data underwent preprocessing to ensure accuracy and consistency. This involved checking for outliers, handling missing values through imputation where appropriate, and verifying the data integrity against entry errors.

Descriptive Statistics: We computed descriptive statistics to summarise the demographic and prescription data. This included calculating the means, standard deviations and ranges for continuous variables, as well as frequencies and percentages for categorical variables.

#### Inferential Statistics

⚫ Chi-Square Tests: We applied chi-square tests to examine the associations between categorical variables, such as gender, age groups, and types of prescriptions.

⚫ Analysis of Variance (ANOVA): To compare the means of continuous variables across multiple groups, such as age groups and drug types, we used one-way ANOVA tests.

⚫ Regression Analysis: Logistic regression was utilised to explore the predictors of drug use, adjusting for potential confounders like age, gender, and diagnosis.

Measures of Association: Results from the logistic regression provided odds ratios (ORs) and 95% confidence intervals (CIs), providing insights into the strength and direction of the associations between demographic factors and drug prescription patterns.

Data Visualisation: To aid in the interpretation of the results, we produced various charts and graphs, including histograms for distribution views, and line graphs to depict trends over time.

Significance Levels: All tests were two-tailed, with a significance level set at *p* < 0.05. This threshold was used to determine the statistical significance of the findings.

## 4. Results

### 4.1 Study Population and Groups

Study population: Male (38.2%) and female (61.8%) patients aged 14–80 years old who required the use of BZDs and non-BZDs.

Age group: The groups were ≤17, 18–34, 35–44, 45–64, 65–75, and >75 years of age.

There was a total hospital consumption of 8 different BZD types and 1 type of Z-drug in the 6 years from 2015 to 2021.

A total of 33,569 valid BZD and Z-drug outpatient and emergency prescriptions were collected from 2017 to 2021.

Inclusion Criteria:

Patients who received benzodiazepine or Z-drug prescriptions between 2015 and 2021.

Data from both specialised psychiatric institutions and general hospitals in Zhejiang, Jiangsu, and Shanghai were collected.

Exclusion Criteria:

Incomplete or missing prescription data.

Patients who were not prescribed BZDs or Z-drugs.

Inpatient prescriptions were excluded.

### 4.2 Defined Daily dose Value of Drug Use

**Fig. 1.**
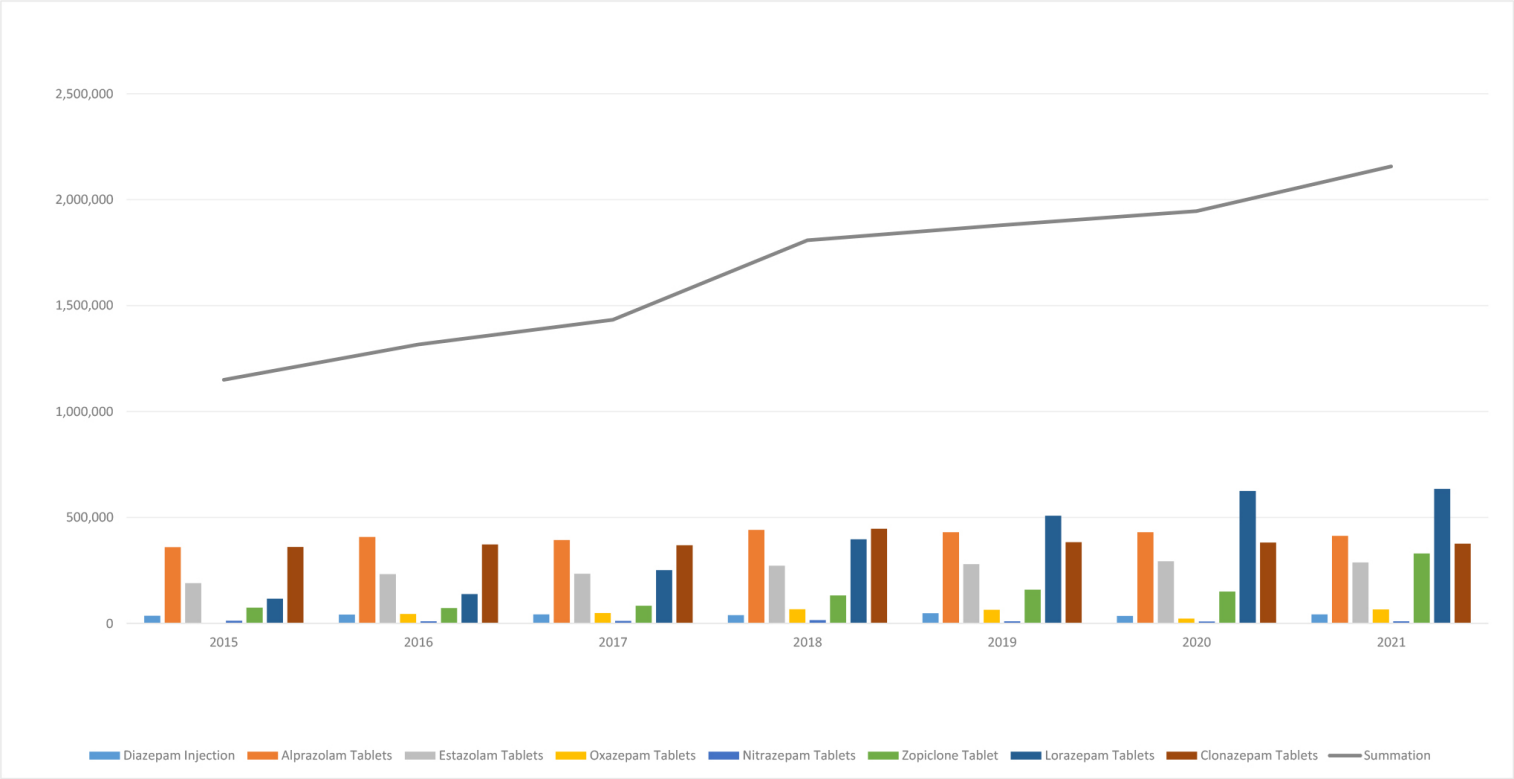
**Trends in benzodiazepines and non-benzodiazepines drug use data (DDDs), 2015–2021**.

The total DDD values of the drugs that were used were calculated according to the general name of each drug, based on the DDD value of said drug, published on the WHO website. The annual DDDs of the total drugs showed an upward trend. Zolpiclone is the primary Z-drug available in the regions where our study was conducted. Eszopiclone and Zolpidem are less commonly prescribed due to regional pharmaceutical regulations and availability. According to the annual DDD value of individual drugs, lorazepam and zopiclone tablets showed a rapid rising trend; oxazepam and estazolam tablets showed a slow rising trend; and alprazolam, clonazepam, and diazepam showed a slight rise, followed by a steady decline. Diazepam and nitrazepam tablets tended to be reduced to non-use. See Table 1 and Fig. 1.

**Table 1.** **In 2015–2021 benzodiazepines and Z-drug use data (DDDs)**.

Drug name	2015	2016	2017	2018	2019	2020	2021
Diazepam Injection	35,631	41,100	42,477	38,900	47,409	35,343	42,008
Alprazolam Tablets	359,120.768	407,427	392,942	440,784	430,069	430,521	412,809.6
Estazolam Tablets	189,380.6667	232,187	234,288	272,133	279,163	292,580	286,880
Oxazepam Tablets	2334.86	43,792	48,936	66,990	63,988	21,841	65,760
Nitrazepam Tablets	12,207	9863	11,341	15,000	9432	8629	9240
Zopiclone Tablet	73,922.04	71,799	83,087	131,808	158,681	150,166	329,952
Lorazepam Tablets	116,098	138,094	251,344	396,718	508,118	624,603.6	634,227.2
Clonazepam Tablets	360,920.8	372,080	368,418	446,312	383,140	381,630	376,038.8
Summation	1,149,615.135	1,316,612	1,432,833	1,808,645	1,880,000	1,945,313.6	2,156,915.6

Note: DDDs, Defined daily doses of the total drugs.

### 4.3 Statistics of Outpatient and Emergency Prescriptions

#### 4.3.1 Population Characteristics of the Drug Users

**Gender**

Of the total number of 33,569 effective prescriptions, 12,836 prescriptions for men accounted for 38.2% and 20,762 prescriptions for women accounted for 61.8%. See Table 2. Among those aged 50–75, 6937 men accounted for 36.7%, and 11,964 women accounted for 63.3%. See Table 2.

**Table 2.** **Characteristics of the population using these drugs**.

Age	Frequency	Male	Valid Percent	Female	Valid Percent
≤17	592	183	30.91%	409	69.09%
18–34	3847	1499	38.97%	2348	61.03%
35–49	6739	2682	39.80%	4057	60.20%
50–64	11,846	4107	34.67%	7739	65.33%
65–75	7055	2830	40.11%	4225	59.89%
>75	3519	1535	43.62%	1984	56.38%
Summation	33,598	12,836	38.20%	20,762	61.80%

**Age**

Among the total 33,569 effective prescriptions, 592 prescriptions for the ≤17 age group accounted for 1.8%, 3847 patients aged 18–34 accounted for 11.4%, 6739 prescriptions for the 35–49 age group accounted for 20.0%, 11,846 prescriptions for the 50–64 age group accounted for 35.2%, 7055 prescriptions for the 65–75 age group accounted for 21.0%, and in the >75 age group, 3519 accounted for 10.5%. There was little difference between the male age ratio and the total age ratio.

**Primary diagnosis and age**

**Insomnia and sleep disorders**: There were 33,569 effective prescriptions, 12,684 of which were diagnosed as insomnia and sleep disorders, accounting for 37.7%. Among the samples diagnosed with insomnia and sleep disorders, 42.3% were men and 57.7% were women. Insomnia and sleep disorders accounted for 41.7% of the prescription statistics for men and 35.2% of the prescription statistics for females. Among the age groups with insomnia, 71.4% were over the age of 50. The 18–34 age group accounted for 8.2%, and the 35–49 age group accounted for 19.8%. The ≤17-years age group accounted for 0.5%.

**Anxiety disorders**: The total number of effective prescriptions was 33,569, 9910 of which were diagnosed as being anxiety disorders (29.4%). Among the sample of patients diagnosed with anxiety disorder, 34.3% were men and 65.7% were women. Anxiety disorders accounted for 26.4% of prescriptions for men and 31.3% of prescriptions for women. Among all of the age groups, 64.1% were over the age of 50. The 18–34 age group accounted for 18.9%, and the 35–49 age group accounted for 23.0%. The ≤17 age group accounted for 1.4%.

**Depression**: Among the total effective prescriptions, 5620 were for a diagnosis of depression, accounting for 16.7%. In the sample of patients diagnosed with depression, men accounted for 35.1% and women for 64.9%. Depression accounted for 15.3% of the prescription statistics for men, 17.6% for women, 54.3% for those over 50 years of age, 20.4% of the 18–34 age group, 20.6% of the 35–49 age group, and 4.7% for the ≤17 age group.

#### 4.3.2 Drug Composition and Disease Distribution

The total effective prescriptions were 33,569, of which 8849 accounted for clonazepam tablets (26.3%), 9425 for lorazepam tablets (28.0%), 5284 for estazolam tablets (15.7%), 5179 for alprazolam tablets (15.4%) and 3303 accounted for zopiclone tablets (9.8%). Furthermore, 3085 accounted for oxazepam tablets (9.2%), 70 for diazepam tablets (0.2%) and 11 for nitrazepam tablets. The proportion of estazolam and zopiclone tablets for insomnia and sleep disorders was higher, and the proportion of lorazepam tablets for anxiety was higher. See Table 3.

**Table 3.** **Statistical table of commonly used benzodiazepines and Z-drug prescriptions for diagnosis of diseases**.

Drug category	Anxiety disorders	Insomnia, sleep disorders	Depression	Other diseases	Summation	N
Alprazolam Tablets	29.60%	30.10%	19.50%	20.80%	100.00%	5179
Oxazepam Tablets	25%	42.40%	11.60%	20.90%	99.90%	3085
Estazolam Tablets	14%	59.30%	8.00%	18.70%	100.00%	5284
Clonazepam Tablets	24.30%	41.30%	17.20%	17.20%	100.00%	8849
Diazepam Tablets	18.60%	52.80%	1.40%	27.20%	100.00%	70
Nitrazepam Tablets	9.10%	36.40%	0	54.50%	100.00%	11
Zopiclone Tablets	20.50%	54.50%	12.20%	12.80%	100.00%	3303
Lorazepam Tablets	37.80%	21.20%	19.80%	21.20%	100.00%	9428

#### 4.3.3 Annual

Statistics show that from 2017, the proportion of second-class mental health in outpatient and emergency prescriptions rose slowly to 33.10% in 2021. The proportion of second-class mental health showed a slowly increasing trend year by year, and the number of second-class mental health also showed a slowly increasing trend each year as shown in Table 4.

**Table 4.** **Statistical table of outpatient and emergency prescriptions and second-class mental prescriptions in specialized hospitals from 2017 to 2021**.

Annual	Second-class mental prescriptions	Outpatient emergency treatment	Proportion
2017	106,134	356,348	29.78%
2018	97,023	325,565	29.80%
2019	109,745	360,212	30.47%
2020	110,558	334,798	33.02%
2021	115,156	347,856	33.10%

**Fig. 2.**
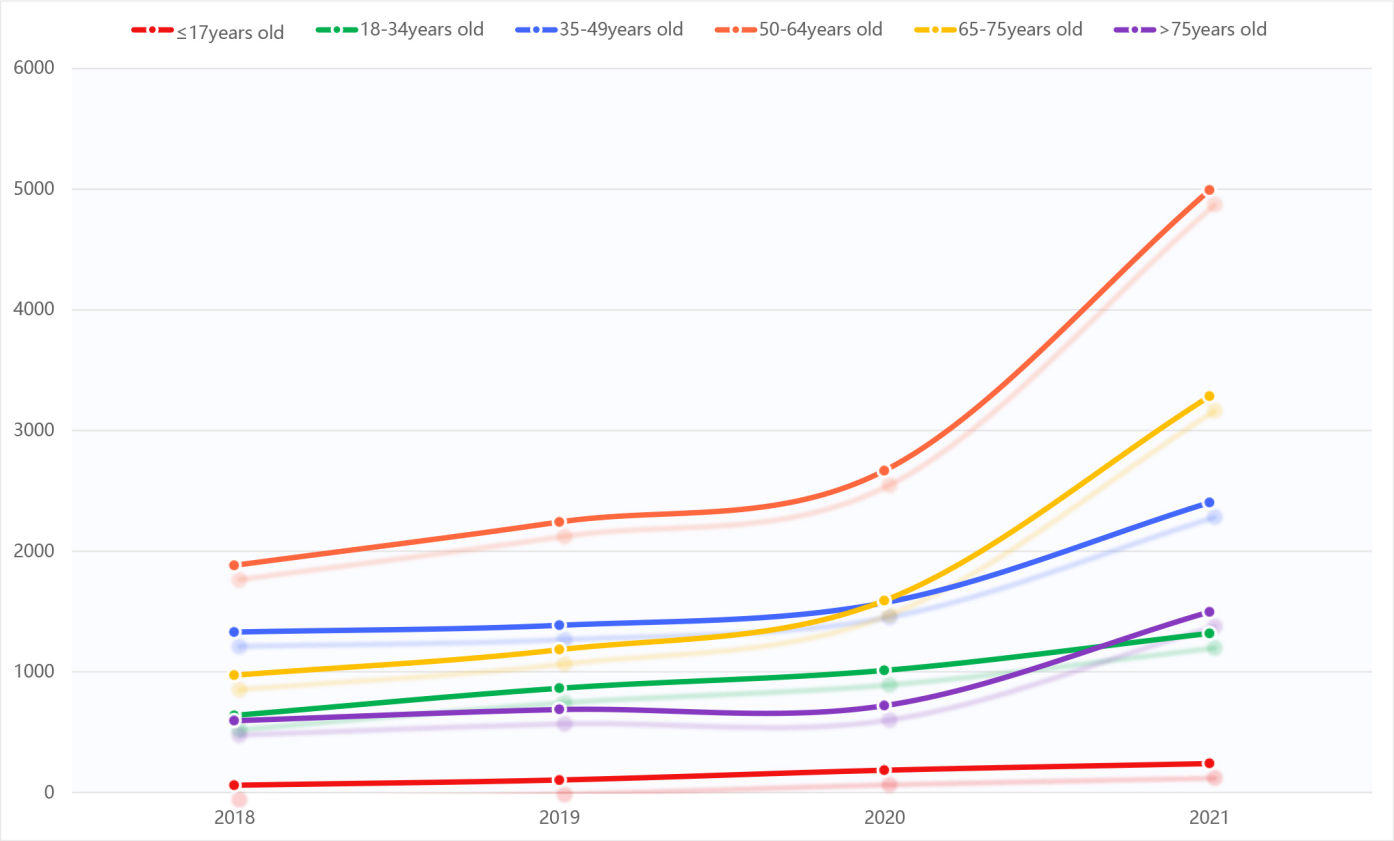
**Summary of sample prescriptions for benzodiazepines and Z-drugs by age group from 2018 to 2021**.

A total of 33,598 valid prescriptions were sampled, 5480 of which had been issued in 2018 (16.4%), 6468 in 2019 (19.2%), 7749 in 2020 (23.1%), and 13,733 were issued in 2021 (40.9%). For 2017, there were only 21 days of data due to a system update (see Table 5). The statistics indicated that the prescriptions of different age groups also showed an increasing trend year by year (see Fig. 2).

**Table 5.** **Summary of sample prescriptions for benzodiazepines and Z-drugs by age group from 2018 to 2021**.

Annual	≤17 years old	18–34 years old	35–49 years old	50–64 years old	65–75 years old	>75 years old
2018	61	639	1330	1883	972	595
2019	104	864	1386	2242	1184	688
2020	185	1012	1573	2668	1591	720
2021	240	1319	2403	4991	3283	1497
Summation	590	3834	6692	11,784	7030	3500

#### 4.3.4 Prescribing Patterns between General and Psychiatric Hospitals

Significant differences in prescribing patterns were observed between hospitals and psychiatric hospitals, while the differences among psychiatric hospitals were not notable (see Table 6, Ref. [12]).

**Table 6.** **Percentage of prescriptions for benzodiazepines and Z-drugs in psychiatric hospitals and general hospitals’ outpatient and emergency departments**.

Type of hospital	Average ratio of benzodiazepine and Z-drug prescriptions to total prescriptions in outpatient and emergency departments (%)	Range	Annual
Regional specialized psychiatric hospital	31.23%	29.78%–33.10%	2017–2021
Five large general hospitals in the 1990s	4.16%	1.32%–7%*	1997–2016

*There are no significant differences in the follow-up [12].

#### 4.3.5 Prescription Frequency and Monitoring Trends from 2017 to 2021

From 2017 to 2021, prescription frequency data-based on patient IDs showed that the highest frequency was 27 times for 1 patient (see Table 7). Most prescriptions were for intermittent rather than long-term use, with fewer cases of repeated prescriptions for the same patient. This trend may be due to the new hospital system implemented after 2017, which included special monitoring and limitations on certain medications. Additionally, continuous monitoring and the regulation of prescriptions, as well as patient education on safe and rational medication use, remain a necessity.

**Table 7.** **Prescription frequency statistics for patient name from 2017 to 2021**.

		Frequency	Percentage	Effective percentage	Cumulative percentage
Effective	1	20,106	78.6	78.6	78.6
	2	3967	15.5	15.5	94.1
	3	942	3.7	3.7	97.7
	4	294	1.1	1.1	98.9
	5	111	0.4	0.4	99.3
	6	42	0.2	0.2	99.5
	7	14	0.1	0.1	99.5
	8	53	0.2	0.2	99.7
	9	38	0.1	0.1	99.9
	10	1	0.0	0.0	99.9
	11	4	0.0	0.0	99.9
	12	4	0.0	0.0	99.9
	16	8	0.0	0.0	100.0
	17	3	0.0	0.0	100.0
	18	6	0.0	0.0	100.0
	27	1	0.0	0.0	100.0
	Total	25,594	100.0	100.0	

## 5. Discussion

### 5.1 Dosage 

There is growing concern about the potential for misuse/abuse, dependence, and addiction to BZD drugs, which are increasingly being prescribed in developed countries. Benzodiazepines were used in 16.6 million of 133.3 million outpatient visits, and in 1.9 million of 18.1 million emergency department visits from 2001 to 2010 in the United States [13]. In 2018, more than 47 million BZD prescriptions, and more than 14 million Z-drugs prescriptions were written in the United States alone [14]. Long-term BZD prescriptions in Australia increased from 4.4% in 2011 to 5.8% in 2015, with a relatively stable annual growth rate of +2.5% between 2015 and 2018 [15].

There were more than 50 million health care consultations between 2011 and 2018 [16]. The prescriptions of BZDs or Z-drugs in Ireland increased from 11.9% in 2005 to 15.3% in 2015 [17]. From January 2017 to December 2020, 1.442 billion BZDs were dispensed across Canada [18].

This study found that the annual DDDs of BZDs and Z-drugs showed an upward trend. Furthermore, the proportion of second-class psychiatric prescriptions in outpatient and emergency prescriptions rose slowly from 29.78% in 2017 to 33.10% in 2021, and the proportion of second-class psychiatric prescriptions and the number of second-class psychiatric prescriptions showed a slowly increasing trend year by year.

### 5.2 Age 

Expanding on these initial observations, BZDs were used by 16% of elderly patients in Australia’s 65-year-old population [19]. Older patients were six times more likely than younger patients to be on long-term BZD prescriptions. American adults aged 50 years and older were more likely than younger adults to use BZDs more frequently than prescribed [13], and 13.75% of elderly patients in the general hospitals of large cities in Germany were dependent on BZDs and Z-drugs [14]. A study on the correlation between sleep status and frailty in Chinese adults aged 30–79 years found that insomnia disorder was associated with an increased proportion of frailty [9]. This study showed that the prescriptions in different age groups also showed an increasing trend year by year, and the increasing trend was larger in 50–64 and 65–75-year-olds than in other age groups.

### 5.3 Women

A total of 38% of women aged >80 years of age in France used BZDs [20]. The 10-year trends in the use of sleeping pills in Switzerland found that, overall, women, the elderly, and people with a history of anxiety and depression were more likely to take sleeping pills. Long-term BZD prescriptions are more common and last longer in older women in Australia [15]. Women in the United States are about twice as likely as men to be prescribed BZDs, according to a press release from the National Institutes of Health on 17 December 2014. The percentage of women using BZDs and Z-drugs in this study was 61.8%. Among them, 63.3% were women between 50 and 75 years old. The number of BZD and non-BZD prescription drug users is much higher in women than in men, based on gender, economic factors, and social and family roles.

### 5.4 Disease

Considering the above demographic trends, BZDs are the main therapeutic drugs for patients with anxiety, insomnia, and epilepsy, and also serve as important adjunctive drugs for patients with specific physical and severe mental diseases to improve insomnia, anxiety, agitation, and other symptoms. In this study, the main disease factors for using BZDs and Z-drugs were anxiety disorder (24.3%), insomnia (41.30%), and sleep disorder (17.2%). Statistics showed that the main disease factors of BZDs and Z-drugs were also different in different age groups. Insomnia and sleep disorders accounted for 71.4%, and anxiety disorders accounted for 64.1% of people over 50 years old. Among the 35–49 age group, 19.8% had insomnia or sleep disorders, 23.0% had anxiety disorders, and among the 18–34 age group, 8.2% had insomnia or sleep disorders and 18.9% had anxiety disorders.

### 5.5 Adverse Reactions

With these clinical implications in mind, various adverse events caused by or related to the use of BZDs and Z-drugs have been widely reported [21]. Furthermore, there is an increased likelihood of falls and delirium associated with the long-term use of BZDs in older adults [22], and an increased risk of cognitive decline, fractures, and episodes of drug dependence in older patients [23]. Benzodiazepine users aged 85 and older fall more frequently [13].

### 5.6 The Non-Medical Use of BZDs and Z-Drugs has the Potential for Addiction and Dependence

These drugs are controlled under various international drug control treaties, according to the Global Drug Report 2018 of the United Nations Office on Drugs and Crime [5]. Benzodiazepines are the most commonly abused type of prescription drug for non-medical use and have become a growing problem. The non-medical use of BZDs is the most prevalent in the UK among illegal stimulants [24]. Non-medical use, including BZDs, is also growing in the Asia–Pacific region, including China [25]. Appropriate interventions are needed to address prescription drug abuse, including BZDs.

### 5.7 Pandemic

This study includes data before and up to approximately two years following the onset of the COVID-19 pandemic. The pandemic may have impacted the study, especially since these medications treat many mental health conditions that were exacerbated by the psychosocial effects of the pandemic. The authors analysed the data, and the histogram from the prescription showed no differences in 2020 compared with 2019 and a small difference in 2021 compared with 2020.

### 5.8 Importance

In synthesising the insights provided by the current research results, this study provides critical insights into the prescribing trends of BZDs and Z-drugs in China, revealing significant increases in usage that bear implications for both clinical practice and public health. Our findings highlight a substantial reliance on these medications, particularly among elderly populations, underscoring an urgent need for policy interventions. By documenting demographic and drug-specific trends, this research contributes to a better understanding of the potential risks associated with long-term use, including dependency and adverse effects. The data serves as a foundation for developing targeted guidelines that can enhance patient safety and optimise prescribing practices. This is crucial in settings where ageing populations are particularly vulnerable, and where there is a need to balance therapeutic benefits against the risks of harm.

### 5.9 Conformance with Zhejiang Provincial Hospital’s Guidelines

Our study aimed to evaluate the prescription patterns of benzodiazepines (BZDs) and their alignment with the Zhejiang Provincial Hospital’s guidelines. The findings indicate that prescribed dosages, such as 5 mg/day for diazepam and 1.5 mg/day for lorazepam, were within recommended ranges. Diagnoses primarily included anxiety disorders and insomnia, consistent with the hospital’s criteria. Additionally, 80% of BZD prescriptions adhered to the short-term use guideline of 4–6 weeks, with exceptions clearly documented. These results demonstrate a high degree of conformity with the hospital’s guidelines, reflecting cautious and appropriate prescribing practices. Overall, our data underscore the effectiveness of these guidelines in ensuring the safe and appropriate use of BZDs, with dosages, diagnoses, and treatment durations generally meeting the recommended standards.

## 6. Limitations of this Study

This study has a few limitations. The retrospective design may include some data inaccuracies, despite our efforts to ensure accuracy. The geographic focus on Zhejiang, Jiangsu, and Shanghai limits generalizability to other regions. Additionally, excluding over-the-counter and private clinic prescriptions might underestimate overall drug use. Lastly, the study period from 2015 to 2021 does not capture longer-term trends. Future research should address these areas to provide a more comprehensive view. Despite these limitations, our findings offer valuable insights into BZD and Z-drug prescription trends in Eastern China.

## 7. Conclusions

The use of addictive BZDs and non-BZDs showed an increasing trend year by year, and the use of addictive BZDs and non-BZDs showed an increasing trend in age. The elderly population, notably, the elderly female population, had a higher proportion of drug use; accordingly, drug safety measures for this population require more attention.

Extrapolation: Management of the rational and safe drug use of BZDs and non-BZDs should be strengthened to reduce the safety risks caused by long-term irrational use. Targeted interventions are needed to reduce the potentially inappropriate long-term prescription and use of these drugs.

## Availability of Data and Materials

The datasets used and/or analyzed during the current study are available from the corresponding author on reasonable request.
